# Organic transistor platform with integrated microfluidics for in-line multi-parametric *in vitro* cell monitoring

**DOI:** 10.1038/micronano.2017.28

**Published:** 2017-08-14

**Authors:** Vincenzo F. Curto, Bastien Marchiori, Adel Hama, Anna-Maria Pappa, Magali P. Ferro, Marcel Braendlein, Jonathan Rivnay, Michel Fiocchi, George G. Malliaras, Marc Ramuz, Róisín M. Owens

**Affiliations:** 1 Department of Bioelectronics, Ecole Nationale Supérieure des Mines, CMP-EMSE, MOC, 880 Avenue de Mimet, Gardanne 13541, France; 2 Flexible Electronics Department, Ecole Nationale Supérieure des Mines CMP-EMSE, MOC, 880 Avenue de Mimet, Gardanne 13541, France

**Keywords:** bioelectronics, in-line sensors, *in vitro*, microfluidics

## Abstract

Future drug discovery and toxicology testing could benefit significantly from more predictive and multi-parametric readouts from *in vitro* models. Despite the recent advances in the field of microfluidics, and more recently organ-on-a-chip technology, there is still a high demand for real-time monitoring systems that can be readily embedded with microfluidics. In addition, multi-parametric monitoring is essential to improve the predictive quality of the data used to inform clinical studies that follow. Here we present a microfluidic platform integrated with in-line electronic sensors based on the organic electrochemical transistor. Our goals are two-fold, first to generate a platform to host cells in a more physiologically relevant environment (using physiologically relevant fluid shear stress (FSS)) and second to show efficient integration of multiple different methods for assessing cell morphology, differentiation, and integrity. These include optical imaging, impedance monitoring, metabolite sensing, and a wound-healing assay. We illustrate the versatility of this multi-parametric monitoring in giving us increased confidence to validate the improved differentiation of cells toward a physiological profile under FSS, thus yielding more accurate data when used to assess the effect of drugs or toxins. Overall, this platform will enable high-content screening for *in vitro* drug discovery and toxicology testing and bridges the existing gap in the integration of in-line sensors in microfluidic devices.

## Introduction

In the near future, preclinical testing of new drugs using *in vivo* animal models is expected to be entirely substituted by less expensive, predictive multi-parametric *in vitro* cell culture models^1–3^. Currently, the most common *in vitro* models are still based on static cell culture models. Although these models have made possible significant advancements in biological research^4^, they have intrinsic limitations due to inadequate mimicking of the cell microenvironment of tissues and organs, thus inaccurately representing cell–cell and cell–ECM (extracellular matrix) communications as well as mechanical and biochemical cues^5^. To overcome these limitations, alternative approaches are offered by three-dimensional (3D) cell cultures^6^ and, more recently, by microfluidics organ-on-a-chip technology^7^.

The organ-on-a-chip field has witnessed remarkable progress in the past few years^8^. The emergence of organ-on-a-chip technology provides a valuable new approach to finely mimic functional units of a specific organ using perfusable micron-sized microfluidic devices. Several examples of organ-on-chip devices have already been described, such as a lung-on-a-chip array^9^, a human kidney proximal tubule-on-a-chip^10^, and a multi-organ-on-chip device platform for the co-culture of intestine, liver, skin, and kidney models^11^. The field is fast moving toward the development of novel and more complex microfluidic devices to host these organoid/tissue models^12–18^; however, few existing research efforts have focused on the integration of in-line sensors, for example, monitoring of cell metabolites, or transepithelial resistance (TER), within the microfluidic environment, while maintaining compatibility with optical monitoring, despite the perceived demand^19,20^.

In-line monitoring systems are in high demand for integration with *in vitro* cell culture models, particularly for organ-on-a-chip devices^7,21,22^. The coupling of in-line sensors with classical biological methods can have a tremendous impact on the future advancement of the field, due to the access to real-time information, without losing the ability to carry out end-point assays. The use of in-line sensors with *in vitro* cell models can have a deep impact on the understanding of cell differentiation, proliferation, dynamics, and indeed functionality under normal conditions and when stimulated/challenged with external mechanical and (bio)chemical cues. When comparing discrete assays, for example, permeability assays, live cell imaging, or reporter assays, with in-line monitoring systems, their twin limitations are the lack of temporal resolution and the use of tags or probes. The former results in the loss of useful information on dynamic changes that may occur in the system under investigation, while the latter is both restrictive in terms of available reagents, and may generate artefacts related to the tag/probe.

As a means of solving these issues, the emerging field of organic bioelectronics^23,24^ gives access to unique tools for label-free, real-time sensing that can potentially bridge the existing gap between rigid, difficult to integrate transducers and soft, architecturally complex tissues. Of particular interest at this interface is the organic electrochemical transistor (OECT), a class of organic devices comprising a thin layer of a conducting polymer as the active material^25^. OECTs are three-terminal devices (source, drain, and gate) in which the conducting layer is deposited between source and drain, forming the channel of the transistor. The transistor channel is typically in direct contact with an electrolyte within which a gate electrode is also present. Poly(3,4-ethylene-dioxythiophene):poly(styrene sulfonic acid) (PEDOT:PSS) is a conducting polymer that is commonly employed as the active layer of OECTs, due to its easy processability, chemical tunability, and biocompatibility^26–28^. Solution processability of this material implies a flexibility of design essential for integration of devices with state of the art *in vitro* models, and indeed, incorporation of microfluidics. PEDOT:PSS OECTs have been fabricated on a variety of substrates, including conformable ones, for interfacing with tissues *in vivo*^29^. The working principle of the OECT relies on the direct electrochemical doping/de-doping of the PEDOT:PSS from ions in the electrolyte entering into the active area, upon application of a gate bias (<0.5 V), thus serving as an efficient ion-to-electron transducer. OECTs have been employed in a wide range of applications, ranging from chemo/bio-sensing^30,31^, to *in vivo* brain activity recording^29^ and *in vitro* measurements of barrier tissue integrity^32,33^ or electrogenic cells^34^. Similar to the commercially available cell-based impedance sensing systems (ECIS, xCelligence), OECTs operating in the AC regime (1 Hz⩽ƒ(Hz)⩽20 kHz) can also provide information on two of the most important cell electrical parameters, resistance and capacitance. OECT technology is also compatible with brightfield and fluorescence high-resolution microscopy as the PEDOT:PSS active layer is optically transparent^35^. Notably, the OECT has also been demonstrated for highly sensitive and specific metabolite sensing from complex media, through biofunctionalization of the gate electrode^26,30,36–38^.

Here we demonstrate for the first time the coupling of OECTs with microfluidics to achieve a multi-parametric transducer platform as a means to study *in vitro* models when cultured inside microchannels. We demonstrate that the microfluidics can be easily fabricated and combined with the OECTs and that laminar flow can be used to apply a mechanical shear stress to the cells. In addition to this, we show that the OECTs can monitor changes in the cell layer capacitance and resistance during flow over an extended period of time, both under normal conditions and when exposed to toxins. Moreover, we use the OECT to perform a fully automated electrical wound-healing assay within the microfluidic device. Combined, this multi-parametric monitoring of live cells in a physiologically relevant fluidic environment offers great promise for *in vitro* toxicology monitoring of live cells.

## Materials and methods

### OECT fabrication and operation

Thermally evaporated gold source, drain, and gate contacts of the OECT were defined on a microscope glass slide via liftoff lithography. Transistor channels of 50×50 μm, 100×100 μm, or 200×200 μm and gate electrodes (2×2 mm) were patterned using a parylene C (PaC) peel-off technique as described previously^39^. PEDOT:PSS (Heraeus, Clevios PH1000, Hanau, Germany) conducting polymer was used as the active layer for the OECT channel and gate electrode. The conducting polymer formulation consisted of PEDOT:PSS, ethylene glycol (Sigma-Aldrich, St. Louis, MO, USA, 0.25 mL for 1 mL PEDOT:PSS solution), 4-dodecylbenzenesulfonic acid (DBSA, 0.5 μL mL^−1^) and 3-glycidoxypropyltrimethoxysilane (GOPS) (10 mg mL^−1^). Before use of the OECTs, the microscope glass slides were soaked in de-ioniwed (DI) water overnight to wash away any unbound material from the OECT channel and gate.

For the operation of the OECTs, a National Instruments PXIe-1062Q system was employed. A source-measurement unit NI PXIe-4145 was used to bias the channel of the OECT (*V*_DS_), while gate potential was applied and controlled using an NI PXI-6289, a multifunction data acquisition module. For frequency-dependent measurements, output currents of the drain (*I*_d_) and the gate (*I*_g_) were recorded using two NI-PXI-4071 digital multimeters. The bandwidth measurements were performed by applying a sinusoidal modulation at the gate electrode (Δ*V*_gs_=20 mV peak-to-peak, 1 Hz<*f*<20 kHz) while keeping a constant bias at the drain (*V*_DS_=−0.4 V). Measurements parameters were controlled using a customized LabVIEW program. For fitting of the frequency-dependent measurements, a MATLAB script was used to extract the cell layer resistance and capacitance as reported previously^40^.

### Microfluidic fabrication and operation

A multilayer approach for the fabrication of the microfluidic device was employed. Briefly, the microfluidic structure was designed with freeware software (CleWin5) and fabricated using a cutting plotter (Large Flatbed plotter – FC2250 series) in order to define the microfluidic channel in a 160 μm thick pressure sensitive adhesive (PSA) (AR8890, Adhesives Research, Limerick, Ireland). By peeling off one of the protective layer of the double-sided adhesive, the PSA was permanently bonded to the substrate by application of constant pressure over the tape. Subsequently, the substrate was plasma activated (25 Watt, 2 min) and a 0.3 mg mL^−1^ collagen type I from rat tail solution was drop cast on the substrate. The substrate was then incubated for 30–45 min at 37 °C in a convection oven to induce crosslinking of the collagen and physical adsorption of collagen fibers on the substrate. Physically adsorbed collagen on the plasma-activated surface promotes the adhesion of cells during cell seeding. At the end of the incubation time, the substrate was gently rinsed with warm (37 °C) de-ionized (DI) water and blown dry with nitrogen.

Following collagen functionalization, the PSA microchannel was permanently sealed using a third layer of a 50 μm thick PMMA (poly (methyl methacrylate), GoodFellow SARL, Lille, France) in which inlet and outlet holes were previously obtained with a 2 mm diameter biopsy punch. For the fluidic connections, inlet and outlet ports were incorporated using a silicone rubber (FDA-compliant silicone rubber adhesive back, 1/4′′ thick).

Before cell seeding, the fully assembled microfluidics, comprising tubing and connection ports, was sterilized with a PenStrep solution (150 U mL^−1^ penicillin, 150 μg mL^−1^ streptomycin, pH 7.4, 1× phosphate-buffered saline (PBS)) for 4 h and then rinsed with fresh Dulbecco’s modified eagle’s medium (DMEM) media. Throughout the experiments, a syringe pump was employed to achieve continuous perfusion of media in the microchannel. Experiments were performed in an XL humidified incubator from PECON GmbH mounted on a microscope Axio Observer Z1 (Carl Zeiss MicroImaging GmbH, Oberkochen, Germany).

### Cell culture

MDCK II (Madin-Darby canine kidney cells from the distal tube of the nephron) were cultivated in DMEM (advanced DMEM reduced serum medium 1, Invitrogen) with 2 mM glutamine (Glutamaxt-1, Invitrogen, CA, USA), 10% fetal bovine serum (FBS) (Invitrogen), 0.5% PenStrep (PenStrep 100, Invitrogen), and 0.1% Gentamicin (Gentamicin 100, Invitrogen). For actin cytoskeleton fluorescent-labeled MDCK II-pLifeAct, transfected with p^CMV^ LifeAct–TagRFP (ibidi GmbH, Martinsried, Germany), the same cell culture media was used, also including 100 μg mL^−1^ of geneticin (Invitrogen).

For cell seeding, cells grown at ~80% confluency in a cell culture flask were incubated with trypsin (0.25%) at 37 °C for 10–15 min. The collected cells were resuspended in fresh DMEM media to a final concentration of ~5×10^6^ cells mL^−1^. Cells were then seeded inside the microfluidic channel and let adhere for 30–45 min. Fresh media was then flowed in the microfluidics in order to rinse away the excess of cells. After seeding, cells were cultivated under constant flow (1.6 μL min^−1^) for 3 days in a humidified 5% CO_2_ incubator. On day 3, MDCK II cells form a confluent barrier with suitable barrier properties (tight junctions). This corresponds to an OECT impedance spectrum having a characteristic cutoff frequency of ~30–40 Hz (Supplementary Figure S1). More details can be found in the Supplementary Information.

### Flow shear stress mechanical stimulation

MDCK II and MDCK II-pLifeAct cells were exposed to flowing culture medium at a fluid shear stress of 0.3 dyne cm^−2^ for 15 h using a syringe pump. Fluid shear stress was calculated using the equation *τ*=6 *μQ*/*bh*^2^, where *τ* is the shear stress, *μ* is the medium viscosity (g cm^−1^ s^−1^), *Q* is the volumetric flow (cm^3^ s^−1^), *b* is the channel width (cm), and *h* is the channel height (cm). The microfluidic device was securely fixed on the stage of a fluorescence microscope. Brightfield and fluorescence pictures of the barrier were taken every 30 min, while an OECT impedance spectrum was recorded every 90 min while a constant shear stress of 0.3 dyne cm^−2^ was applied. Following the 15 h of physiologically relevant flow stimulation, cell layers were kept in culture for a maximum of 6 h before fixation of the cells on the substrate.

### OECT glucose sensor functionalization and sensing

To perform cell glucose uptake quantification using the microfluidics effluent, an OECT was used as transducer and amplifier for enzymatic determination of glucose. Typically, 100 μL of the exhausted media from the microfluidics was collected (every ~1 h) in a Eppendorf tube for 3 h prior and 6 h after application of a physiological relevant fluid shear stress. With regards to the enzymatic functionalization of the OECT, a PEDOT:PSS planar gate electrode (600×600 μm^2^) was modified with glucose oxidase enzyme based on a previously reported method^30^. Briefly, the gate electrode was plasma activated (25 W, 2 min) to favor covalent attachment of a heterobifunctional silane (GOPS) via condensation reaction. Subsequently, a glucose oxidase/chitosan–ferrocene (CS–Fc) complex was then immobilized on the GOPS functionalized surface via EDC/NHS chemistry. CS–Fc acts herein as an electrochemical mediator, thereby enabling electron transport between the enzyme’s active site and the electrode. Before the measurements, a standard calibration curve was obtained by using different dilution ratios of PBS/fresh DMEM media (Supplementary Figure S2). More details can be found in the Supplementary Information.

### Cytochalasin D assay

A confluent MDCK II-pLifeAct cell layer grown inside the microfluidic channel was exposed to 2 mg mL^−1^ cytochalasin D (Cyt D) while recording impedance spectra and brightfield and fluorescent images simultaneously. Typically, 100 μL of fresh DMEM media containing 2 mg mL^−1^ Cyt D was allowed to flow inside the microfluidic device from the outlet of the microfluidic and interact with the cell layer for ~20 min in static condition. Following this time, Cyt D free DMEM media was perfused through the microfluidics and recovery of barrier properties was monitored for *ca.* 2 h.

## Results and discussion

### Flow shear stress mechanical stimulation

The vast majority of the microfluidic platforms published in literature are fabricated using standard ‘soft lithography’, a technique based on the fabrication of a silicon mold as a means to shape a silicone rubber polymer, that is, poly-dimethylsiloxane (PDMS), with micrometer sized features^7^. Subsequently, PDMS is permanently bonded to a flat and smooth substrate, such as glass, following O_2_ plasma activation. As a result, irreversible adhesion of the PDMS microfluidic device is achieved and the device can support long-term operation for days and even weeks.

In the present work, the fabrication of planar OECTs, with both the transistor channel and the gate electrode patterned onto the substrate, is performed on conventional microscope glass slides through liftoff lithography (more details are available in the ‘Experimental’ section). As part of the OECT fabrication, we make use of a thin layer, ~2 μm, of PaC to insulate the gold contact and define the active area of the transistor. PaC is one of the many derivatives of poly(p-xylylene) presenting high mechanical and chemical stability and it has also found many biological applications^41^. However, the presence of PaC as the terminal layer of the substrate poses severe limitations on the fabrication of PDMS microfluidic devices using standard soft lithography. In fact, due to the different chemical structures of PDMS and PaC, it is not possible to achieve permanent bonding of these two substrates by simple O_2_ plasma activation. The poor adhesion between the two substrates results in leaks and, eventually, delamination of the PDMS structure from the substrate. Some strategies have been proposed for the irreversible bonding of PDMS and other plastic substrates^42,43^, however, on the basis of our initial experiments, it was not possible to achieve stable adhesion of PDMS and PaC using these protocols. A possible solution to this can be the complete removal of the PaC layer from the substrate, making the glass substrate available for O_2_ plasma-mediated bonding with PDMS. However, from an electronic standpoint, the presence of PaC as the insulator layer is essential for operation of the OECTs. Indeed, in the absence of PaC, the electrolyte would also be in direct contact with the source and drain gold contact lines causing additional capacitive effects that in turn will affect the efficiency of the transistor, as shown in Supplementary Figure S3.

**Figure 1:**
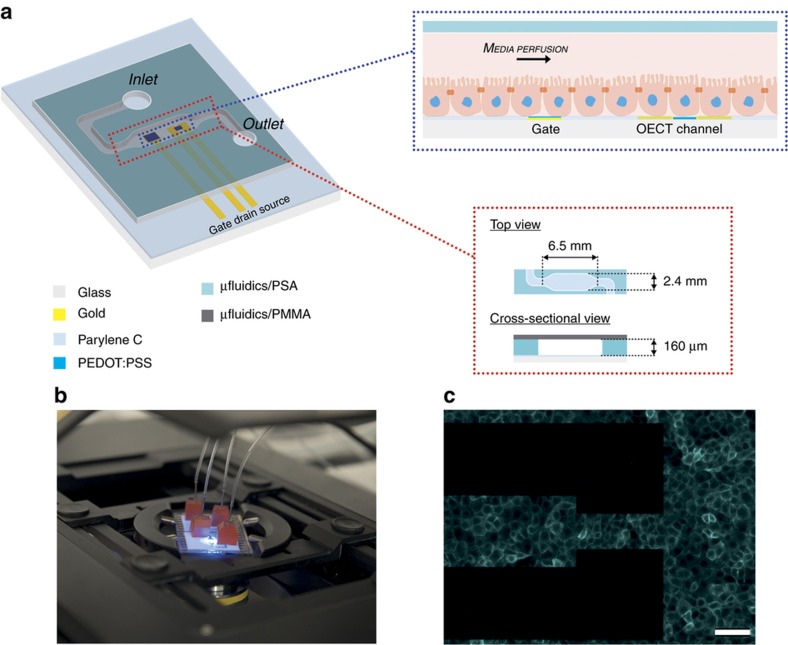
The integration of microfluidics with the OECT for combined optical and electronic monitoring. (**a**) Graphical representation of the developed platform integrating the OECT with microfluidics. Top right, an illustration of the OECTs and the cell layer lining the bottom surface of the microfluidic channel. Bottom right, top and cross-sectional views of the microfluidic device. (**b**) A picture of a fully assembled microfluidic platform located on a microscope stage, featuring inlet and outlet ports and tubing. The red silicone blocks are used to guarantee stable connections between the inlet and outlet tubing and the microfluidic platform. (**c**) Fluorescence image of a fully confluent layer of MDCK II cells transfected with pLifeAct (red fluorescent protein-labeled F-actin) grown inside the microfluidic channel integrated with a planar OECT. The black blocks are the source and drain gold transistor channel contact lines (scale bar, 100 μm). OECT, organic electrochemical transistor; MDCK II, Madin-Darby canine kidney cells from the distal tube of the nephron.

On the basis of these initial observations, and bearing in mind that the microfluidics platform developed has to operate in a stable manner for several days, we decided to circumvent the PaC/PDMS adhesion problem by using alternative materials, for example, acrylic plastic, for the fabrication of the microfluidics. Figure 1a (left) shows a schematic illustration of the developed platform integrating the OECT and the microfluidic device. To carry out the fabrication of the microfluidic device, we made use of a medical grade double-sided PSA that provides an irreversible bonding between the PaC insulated OECTs and the microfluidics^44^.

The proposed platform is made of a single microchannel within which multiple OECT channels and gate electrodes of different geometries may be located in the microfluidic chamber (*W*×*L*×*H*=2.4 mm×6.5 mm×160 μm). The illustration in Figure 1a (zoom-top) shows a cross-sectional view of the device, in which the different components are represented, from bottom to top, the glass/PaC substrate with the OECT channel and gate, the epithelium and the media perfusing through the microfluidic chamber. Figure 1b shows a picture of the fully assembled device positioned on the stage of an inverted microscope where the white layer is the PSA and the adhesive red silicone blocks are used for the connection of the microfluidics with the inlet and the outlet tubing. Figure 1c also shows a fluorescence image using live cell imaging (cells expressing an red fluorescent protein (RFP)-tagged actin) of a fully confluent layer of MDCK II grown inside the chamber of the OECT/microfluidic device.

To establish the appropriate conditions for future experiments, we first investigated the effect of physiologically relevant fluid shear stress (FSS) on the MDCK II cells when cultured inside the prepared microfluidic device, having the microfluidic PSA/PMMA irreversible bonded to a PaC-coated glass slide. MDCK II are epithelial cells from the distal part of the nephron in the kidney that, under physiological conditions, are exposed to the continuous flow of the glomerular filtrate, ranging between 0.2 and 20 dyne cm^−2^ ^45^. To carry out this initial study, we made use of MDCK II-pLifeAct transfected cells that present fluorescent F-actin filaments.

**Figure 2:**
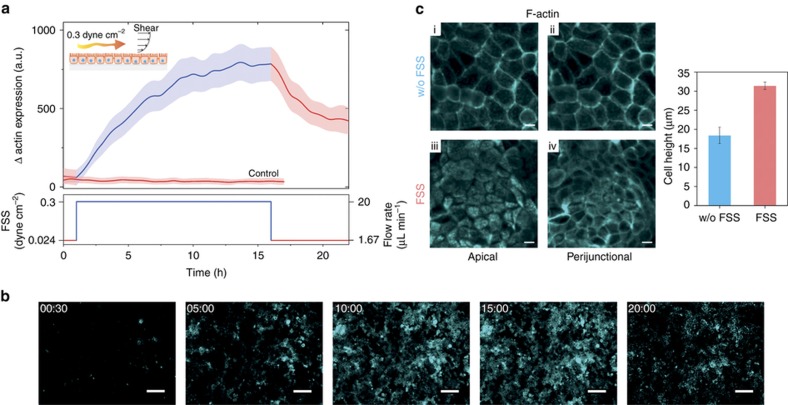
Illustration of the effect of FSS on F-actin expression via time-lapse imaging of cells grown on the platform. (**a**) The variation in the actin expression is shown as the relative increase in the fluorescence intensity (*λ*=584 nm) induced by physiologically relevant fluid shear stress (FSS). A confluent layer of epithelial cells (MDCK II-pLifeAct) are grown to confluency under dynamic conditions with a flow rate equal to 1.67 μL min^−1^, until cells show a typical cobblestone-like morphology. Once the epithelium is fully confluent, a greater flow rate of 20 μL min^−1^ is applied, resulting in an FSS of 0.3 dyne cm^−2^, for a total of 15 h of mechanical stimulation of the cells. Below, the FSS/flow rate profile used for the experiment is represented. (**b**) Fluorescence images of MDCK II epithelium captured at different time (hh:mm) points, before (00:30), during (05:00, 10:00, and 15:00) and after (20:00) application of a flow rate equal to 20 μL min^−1^ (scale bar, 100 μm). (**c**) F-actin confocal images of the apical side (i, iii) and perijunctional section (ii, iv)of a confluent cell layer captured 15 h after exposition at flow rates of 1.67 μL min^−1^ (i, ii) or 20 μL min^−1^ (iii, iv), the latter corresponding to 0.3 dyne cm^−2^ FSS. On the right, cell heights measured by confocal microscopy in *z*-sectioned images showing an increase of the cell height due to the FSS (scale bar, 10 μm).

First, MDCK II-pLifeAct cells were cultured inside the microfluidic device under dynamic conditions at a constant flow rate of 1.67 μL min^−1^ for *ca.* 3 days to reach confluency and a typical cobblestone-like morphology. The constant flow rate guarantees continuous media turnover thus avoiding cell death due to depletion of cell nutrients inside the microchannel^46^. To mechanically stimulate the epithelium with a physiologically relevant FSS, the flow rate was then increased from 1.6 to 20 μL min^−1^. The latter corresponds to an FSS equal to 0.3 dyne cm^−^^2^ (details in the ‘Materials and Methods’ section). These conditions were kept constant for 15 h while fluorescence time-lapse imaging was carried out by capturing an image every 30 min. Interestingly, we observed an increase in F-actin expression (increase in the fluorescence) induced by the application of a continuous FSS. A quantitative evaluation of the increase of fluorescence over time is plotted in Figure 2a (top). As may be appreciated from the graph, cells started to respond to the flow stimulation within 1 h after starting FSS. The change in the actin fluorescence expression becomes more evident over time, reaching its maximum and stabilising after *ca.* 12 h. A decrease in the actin fluorescence was then observed once the flow rate was brought back to its initial value, however, the overall levels of actin expression remained elevated compared to initial values. In contrast, cells kept in culture for the same length of time at 1.67 μL min^−1^ (control Figure 2a) showed no change in actin expression. Time-lapse images taken during the experiment are shown in Figure 2b (see also Supplementary Video 1 from ESI). From this simple experiment, it is clear that cells are sensitive to biomechanical cues, such as an increase in the FSS, inducing substantial changes in expression and organization of the cell cytoskeleton proteins. Here we clearly show in real-time reorganization as well as increased expression of the F-actin filaments by simply providing a more physiologically relevant mechanical shear to the cells. These observations are consistent with previous studies performed by Duan *et al.*^47^, where FSS induced actin reorganization in renal proximal tubule cells. Cyclical induction of the F-actin expression was also observed when cells experienced multiple cycles of increased and decreased flow rates, as shown in Supplementary Figure S4.

In addition to the increase in the F-actin expression, for the FSS-stimulated cells the presence of highly fluorescent F-actin ‘dots’ was observed at the apical side (Supplementary Figure S5iv), as indicated by the red arrows on the zoom inset image, in contrast to the images of either the basal side plus FSS (Supplementary Figure S5iii), or the control cells either basal or apical (Supplementary Figure S5i and ii). Kidney cells lining the tubular lumen of the nephron in the kidney present subcellular microvilli structures to facilitate resorption of salts and nutrients from the glomerular filtrate. Microvilli are characterized by a dense bundle of actin filaments in the core of their structure, that in the fluorescent images are visible as highly fluorescent dots^17,48^. Figure 2Ciii shows a confocal image of the cell layer apical surface after FSS stimulation for 15 h, with discrete areas (bundles) of F-actin clearly visible, indicating well-defined microvilli structures. In contrast to this, the apical side of cells in the absence of FSS stimulation does not present the same morphology, as shown in Figure 2ci. Another significant difference between the two conditions studied is an almost doubling in the cell height (31.43±0.98 μm compared to 18.43±2.15 μm), speaking to a better cell polarization upon application of the FSS. The confocal images allow us to compare actin at the top of the cells (the aforementioned bundles) with the very distinct perijunctional actin visible further down the cell body, (Figure 2Civ). Such differences are not visible in the absence of FSS, (Figure 2Ci and ii).

**Figure 3:**
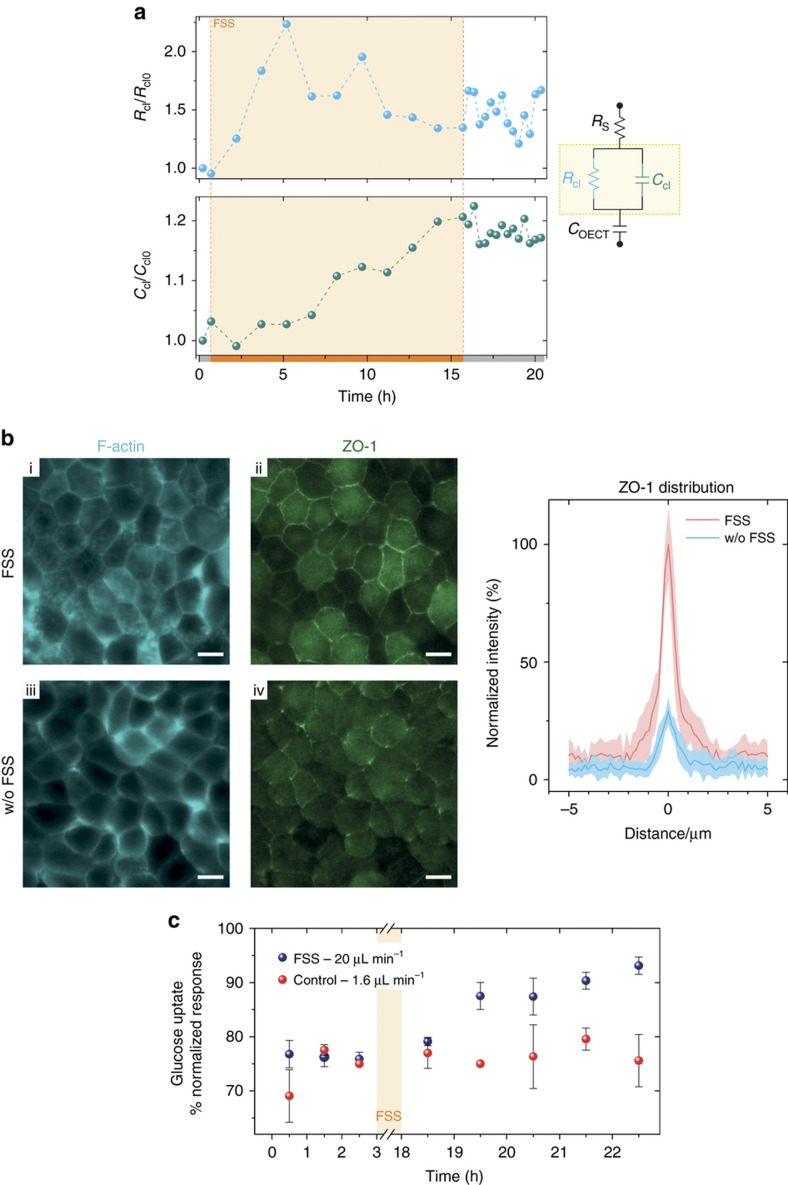
Multi-parametric readout of epithelial cell response to FSS. (**a**) Typical in-line monitoring of the cell layer resistance (*R*_cl_) and capacitance (*C*_cl_) before, during, and after FSS stimulation with a shear stress equal to 0.3 dyne cm^−2^. The cell layer resistance and capacitance was measured every 90 min during the 15 h of constant FSS (orange area on time axis) and every 20 min for 6 h after FSS stimulation (gray area time axis). The inset equivalent circuits highlight the two electrical circuit elements, *R*_cl_ and *C*_cl_, extracted from the fitting of the equivalent circuit model. From the equivalent circuit, *R*_s_ is the series resistance of the electrolyte and C_OECT_ the transistor capacitance (**b**) F-actin and ZO-1 fluorescence images of a confluent cell layer in the presence and absence of FSS stimulation (scale bar, 10 μm). On the right, the fluorescence intensity distribution of ZO-1 tight junction protein with and without FSS is shown (*n*=10). (**c**) Uptake of glucose by the MDCK II cell layer before and after FSS. An increase in the uptake of glucose was observed for cells stimulated with a physiologically relevant shear stress (20 μL min^−1^), while the glucose uptake remains unchanged in the control condition (1.6 μL min^−1^). A sample was collected every 1 h from the microfluidic outlet and glucose content was determined using an OECT-based glucose biosensor (*n*=3). Error bars show standard deviation from the mean of three different samples.

Following these initial results and validation of the microfluidic device, we proceeded to the integration of OECTs as in-line impedance sensors embedded in the microfluidics, to allow electrical monitoring of the optical changes observed. Similar to the previous section, cells are grown under dynamic conditions (flow rate equal to 1.67 μL min^−1^) to reach confluency, showing a typical cobblestone-like morphology. Using the OECT, we could also determine electrically whether the cell layers form a tight barrier by means of a frequency-dependent measurement (Supplementary Figure S1)^33^. Figure 3a shows the temporal evolution of the cell layer resistance (*R*_cl_) and cell layer capacitance (*C*_cl_) while cells were exposed to the FSS, using the protocol described in Figure 2a (bottom).

In parallel with the F-actin fluorescent signal, we also observed an increase in the cell layer resistance with fluctuations up to 2.25-fold from the initial value, stabilizing to *ca. ~*1.5-fold higher after 15 h at 0.3 dyne cm^−^^2^. The resulting increase in the *R*_cl_ can be attributed to the reassembly of intercellular tight and adherent junctions due to FSS-induced mechano-transduction^47^. As further proof of the increase in the paracellular resistance of the cell layer, we performed fluorescence immunostaining of the ZO-1 tight junction protein in order to understand how the reassembly of the intercellular junctions in the cells takes place. Figures 3bii and iv show the ZO-1 fluorescence images captured in the two conditions, when cells are exposed and not exposed for 15 h to the FSS, respectively. Although in both cases, a certain amount of ZO-1 protein is present in the cytosol of the cells, it is clear that the exposure to the FSS induced a significant reorganization of the ZO-1 protein on the borders of the cells. This result suggests that the monitored increase in *R*_cl_ during and after FSS is possibly given by an increase in the density of the tight junction network, thus increasing cell layer resistance *R*_cl_ as observed from the electrical measurement in Figure 3a. A more quantitative analysis of the ZO-1 fluorescence distribution is shown in Figure 3b (right).

The cell layer resistance provides information on the ionic paracellular pathways, while the cell layer capacitance provides indirect information on the size and possibly shape of the cell. In fact, if we assume the cell membrane to be the equivalent of a capacitor insulator, changes in cell membrane area will affect the amount of stored electrical charge. The bigger the cell membrane, the higher the capacitance of the cell layer. In Figure 3a (bottom), the temporal evolution of the cell capacitance for FSS cells is shown. A clear increase of the cell layer capacitance is observed over time with a more stable total increase equal to ~1.15-fold, 1 h after the end of the FSS. As the FSS favors the F-actin reorganization and expression in the cytoskeleton as well as the polarization of the cells, the measured increase in the capacitance may be the consequence of an increase in the cell membrane surface, that is, cell size and microvilli on the epithelium apical surface. The electronic measurement of the cell capacitance is in complete accordance with the formation of microvilli and the increase in the cell height in case of the FSS stimulation, as previously shown in Figure 2c.

With respect to the fluctuations observed in the *R*_cl_ during the FSS stimulation (light orange area, in particular for the peaks at time ~5 and ~10 h), this may be correlated to the application of a mechanical force when a greater fluid flow rate is used, leading to movements and rearrangement of the cell layer on the substrate. Mechanisms to explain these changes in the cell impedance for FSS-stimulated cells may include changes in the cleft height (distance between substrate and cells) and/or paracellular distance of the cells^49^. In the present work, we made use of the equivalent circuit of Figure 3a (right) to model the cell layer covering the transistor channel, adding in series to *R*_s_ (series electrolyte resistance) and *C*_OECT_ (transistor channel capacitance), the cell layer resistance (*R*_cl_) and cell layer capacitance (*C*_cl_) in parallel^40^. Notably, the presence of the cleft height offers an additional ionic resistance (*R*_cleft_) that in our model is counted in the series electrolyte resistance *R*_s_. Taking these points into consideration, we posit that the observed *R*_cl_ fluctuation of Figure 3a (top) is attributed to changes in the cell/cell paracellular distance, due to changes in packing of the cells on the active area of the transistor channel during high flow. In fact, measured changes in the impedance spectra were observed in the frequency range (ƒ(Hz)⩽400 Hz) that are commonly attributed to the paracellular resistance^50,51^. The aforementioned changes in the *R*_cl_ are shown in Supplementary Figure S6, together with the fitting curves used for the extrapolation of *R*_cl_ and *C*_cl_ reported in Figure 3a. It is also important to note that we ascertained that the fluid flow in microfluidics do not adversely affect the OECT performance, as shown in Supplementary Figure S7.

Next, we investigated whether the observed variations in actin expression, resistance, and capacitance were also affecting the glucose transport of the cells. To determine changes in the cells glucose uptake, we first performed a conventional fluorescence immunostaining using a GLUT1 glucose membrane transporter antibody. Differences (see Supplementary Figure S8) in the density or localization of GLUT1 on the cell membrane when comparing FSS-stimulated and -unstimulated cells were not apparent. Nevertheless, we sampled the remaining glucose concentration in DMEM media collected from the microfluidic effluent. This analysis can provide a direct evidence of any changes occurring in the cell metabolism and uptake of glucose induced by the FSS. Taking advantage of the facile biofunctionalization of PEDOT:PSS^26^, we implemented a glucose electrochemical biosensor based on our OECTs technology^52^. Figure 3c shows the glucose uptake of the cells over time, for samples collected before and after the 15 h of FSS stimulation at 0.3 dyne cm^−^^2^. The glucose uptake is calculated as the percentage of the ratio between the final and the initial glucose content in DMEM media (Equation (2) supporting S2). As shown in the graph, there is a clear increase in the glucose uptake for the cells subjected to high media flow (blue/white points) with a maximum of ~95% glucose uptake 6 h after the FSS, compared to the more stable glucose uptake (between ~75 and ~80%) for the control experiment (red/white points). Increased glucose uptake may be related to the expression of multiple transporter types and/or better efficiency of the glucose uptake receptors in the microvilli^53^, and would be consistent with an increased adsorption of nutrients allowed by higher surface area at the apical surface.

### Electrical wound healing and microfluidics toxicity testing

In the previous section, we showed a facile method to achieve integration of the OECTs with microfluidics. One of the key advantages of using the OECT technology for testing the cells is the possibility for easy integration of micron-sized electrodes with a microfluidic structure in a compact manner. As further proof of our capabilities of using the OECTs to perform standard biological assays, we developed an electric wound-healing assay for the cell layer cultured in the microfluidics. In a classic wound-healing assay (or scratch assay), cells are grown in a confluent monolayer and then the layer is ‘wounded’ using a pipette tip or razor blade. Following the formation of the wound, cells in the proximity of the wound are monitored over time using a microscope in order to study their ability to heal and migrate over the damaged area. However, the scratch assay does not offer a precise control on the size and the shape of the wounded area. Moreover, it lacks reproducibility, as the scratch assay is generally a manual technique. To overcome some of these limitations, Keese *et al.*^54,55^ first proposed an alternative method to obtain precise control of the wound size and shape by using AC electrical currents to achieve electroporation of cells (wounding) in a well-defined region corresponding to the area of a micro-sized gold electrode.

Inspired by these findings, we developed an electrical wound-healing assay based on the use of the OECT to generate a wound in the cell layer covering the transistor channel. As mentioned before, in a classical scratch assay the healing of the cells is monitored optically. From a biological stand point, the possibility to visualize the dynamics behind the healing process provides information on the speed and vector of healing (and lead to the elucidation of the purse-string mechanism)^56^. However, one of the main limitations on performing wound-healing assay by electrical means is incompatibility with microscopy as the presence of the gold electrode at the wound site does not allow for the use of an inverted microscope. Conversely, OECT technology is fully compatible with high-resolution microscopy as the PEDOT:PSS active layer of the transistor channel is optically transparent^35^. In this scenario, the use of the OECT to perform an electrical wound-healing assay can provide a unique tool to overcome the current limitations on the use of micron-sized gold electrodes, making this method fully compatible with standard microscopy tools.

**Figure 4:**
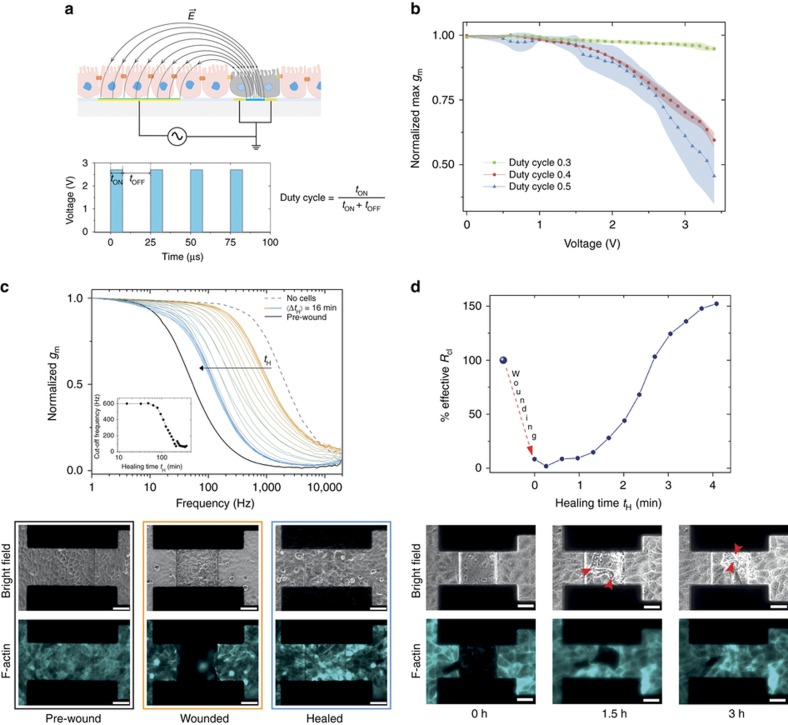
A microfluidic electrical wound-healing assay with the OECT. (**a**) Schematic of the experimental set-up of the developed OECT-based electrical wound-healing assay. A confluent cell layer covering the transistor channel area is electroporated with an oxidative square voltage, typically below 3 V (bottom schematic), resulting in an electrical wound of the same dimension as the transistor channel. The semicircle lines represent the electric field distribution at the electrode/electrolyte interfaces across the cell layer, while the two gray cells covering the transistor channel represent the electrically wounded cells. (**b**) The impact on the OECT maximum transconductance *(g*_m_*=*Δ*I*_D_/Δ*V*_G_) caused by the application of oxidative potentials for duty cycles (see equation) equal to 0.3 (green), 0.4 (red), and 0.5 (blue), *n*=3. (**c**) Typical time evolution of the OECT frequency-dependent response during the healing process of an electrical wound generated on a confluent cell layer of MDCK II-pLifeAct. A confluent layer of cells grown on the transistor channel induces a shift in the OECT cutoff frequency, from ~1400 Hz (dashed gray line) to ~30 Hz (solid black line). Following the generation of the electrical wound (2.7 V at 40 kHz, duty cycle 0.3, cycle time 30 s), the cutoff frequency increases (orange line) due to loss of cells from the active area of the device. As the healing of the cells progresses, a continuous decrease in the cutoff frequency is monitored until completion (blue line). Inset graph shows the sigmoidal evolution in the cutoff frequency during the healing process. The data point at time zero is omitted for clarity. Below are shown brightfield and fluorescence images for the pre-wound (black frame), wounded (orange frame), and healed (blue frame) cell layer (scale bar, 50 μm). (**d**) Electrical wound-healing assay performed inside the microfluidic device. On the top, the temporal evolution of the *R*_cl_ during the healing process is shown. The bottom panel contains the brightfield and the F-actin fluorescence images at different time during the healing process. In the brightfield images, the red arrows highlight the densely packed healing fronts incorporating the wounded cells and likely leading to the final increase of ~1.5-fold in the effective *R*_cl_.(scale bar, 50 μm).

Similar to the electrical wound-healing assay developed by Keese *et al.*, we developed an electrical wound assay using the OECT channel and the gate electrode in a slightly different operation mode compared to the classic transistor configuration^25^, as shown in Figure 4a (top). The source and drain of the transistor are shorted together to obtain an equi-potential distribution of the applied AC voltage in the transistor channel, while a second, bigger, electrode is used to close the electrical circuit and act as the counter electrode for the wound generation. It should be noted that by shorting the source and drain of the OECT channel, effectively an electrode is generated, which, used together with the bigger second electrode (gate electrode), was employed for the generation of the electrical wound in the cell layer. The alternating potential applied to the system is schematically represented in the bottom diagram of Figure 4a. For the generation of the electric wound, a square wave pulse is applied from zero to the desired voltage (typically below 3 V) at a frequency of 40 kHz (period of 25 μs). The schematic of Figure 4a (top) shows the direction of the electric field across the two electrodes in the electrolyte. As the transistor channel is a much smaller electrode, the potential drop at the electrode/liquid interface across the cell layer covering this region is above the critical cell membrane rupturing value (>200 mV)^57^, resulting in a localized cell electroporation and lysis, schematically represented by the gray cells, Figure 4a (top).

For the OECT wound-healing assay, one crucial parameter is the stability of the PEDOT:PSS organic conducting layer upon the application of a high oxidative potential needed to induce the electroporation of the cells. In addition, high potentials (>1.1 V) in an aqueous environment can lead to electrochemical water oxidation and formation of other cytotoxic species, such as chlorine^54^. Interestingly, the use of AC currents at high frequency (>10 kHz) can easily bypass these undesirable conditions even for potentials up to 5 V^55^. Nonetheless, it is well known that the irreversible electrochemical oxidation of PEDOT:PSS-conjugated polymer leads to structural changes of the polymer backbone and loss in its conductivity^58^. Figure 4b shows the variation of the maximum transconductance value of the OECT (*g*_m_*=*Δ*I*_D_/Δ*V*_G_) when sequentially stepping the potential from 0 to 3.4 V at 40 kHz for 30 s each time, using the electrical configuration and the wave signal shown in Figure 4a. By evaluating the variations in the maximum transconductance of the OECT, it is possible to obtain useful information on possible degradation processes induced in the PEDOT:PSS layer. For a duty cycle of 0.5 (blue line/triangle, *t*_ON_=12.5 μs, *t*_OFF_=12.5 μs), a rapid decrease of the OECT transconductance is observed for a potential above 1.5 V, resulting in a total loss of ~50% at 3.4 V. To improve the device stability in this wide potential window, a possible solution is to change the duty cycle of the squared pulse signal. A shorter duty cycle reduces the total supplied energy for undesired oxidative reactions in the conjugated polymer and, at the same time, allows a longer relaxation of the system. For instance, a duty cycle of 0.4 (red line/circle, *t*_ON_=10 μs, *t*_OFF_=15 μs) slightly improves the device stability with a total loss of ~40% at 3.4 V, while for a duty cycle of 0.3 (green line/square, *t*_ON_=7.5 μs, *t*_OFF_=17.5 μs), the OECT shows a more stable behavior in the potential window of interest. In the latter conditions, a final ~6% decrease in the maximum transconductance is observed only when the OECT was sequentially cycled to a potential up to 3.4 V. These findings are particularly important as they provide evidence that under these conditions, that is, 40 kHz, duty cycle 0.3, the OECT channel is capable of supporting high oxidative potentials without an irreversible loss in its amplification performance, an essential requirement to perform in-line electrical monitoring of the cell layer during healing.

Next, we performed the electrical wound-healing assay by seeding MDCK II-pLifeAct cells on the OECT. Initial optimization experiments were performed in a classic, static configuration using a glass well to contain the cell culture media. Figure 4c shows a typical frequency-dependent transistor response in the absence (dashed gray curve) and in the presence of a fully confluent MDCK II-pLifeAct cell layer (black solid line). In the images below (black frame), the brightfield and the F-actin fluorescence pictures of a confluent cell layer are shown. The electrical wound to the cells was then generated by pulsing the electrode with 2.7 V at 40 kHz (duty cycle 0.3) for 30 s. An identical pulsing protocol was repeated four times until a complete absence of fluorescence covering the transistor channel was observed. The F-actin fluorescence images (orange frame) shows the well-defined square shape of the wound corresponding to the size of the transistor channel (100×100 μm^2^), proving the ability of the OECT to create an electric wound in a well-confined area of the cell layer. Following the formation of the wound, we then started to monitor the healing process optically and electronically, by using the same OECT employed for the generation of the wounding. Moreover, the electronic monitoring of the healing process was performed by using the OECT as a three-terminal device. Figure 4c shows the time evolution of the frequency-dependent response of the OECT measured every 16 min while cells were healing, shown by the gradual color change of the curves from orange (wounded cell layer) to blue (healed cell layer). As the healing begins, we measured slight variations in the cutoff frequency arising from the initial rearrangement of the cells in the proximity of the electrical wound, and formation of two moving healing fronts (see Supplementary Video 2 ESI). The initial stage of the healing process corresponds to the closely packed orange curves of Figure 4c. Subsequently, with the advancing of the healing fronts toward the middle of the wound a continuous decrease in the cutoff frequency is observed until completion of the healing, as revealed by the proximity of the blue curves. The electrical evolution of the healing process can be appreciated in more detail in the inset graph of Figure 4c, showing a well-defined sigmoidal trend with two steady state conditions corresponding to the start, with a cutoff frequency equal to ~600 Hz, and the end of the healing, with a cutoff frequency equal to ~60 Hz, respectively. The images of Figure 4c (blue frame) show the brightfield and fluorescence images of the fully healed cell layer.

Encouraged by these findings, we then integrated the OECT electrical wound-healing assay with the microfluidics. Figure 4d shows the typical time evolution of the cell layer resistance obtained during the healing process of the cells. First, the cells were wounded on the transistor channel resulting in a complete loss of cell-related impedance, as well as the actin fluorescence, in a well-defined manner, as shown before and in the inset fluorescence picture at time zero. It is also important to note from the brightfield image that, following electroporation, dead cells were still covering the transistor channel area although they were not responsible for substantial resistive contributions (*R*_cl_<10% of initial value) within the first hour of the healing process. As the healing front of the cells started to move toward the center of the transistor channel, we measured a continuous increase in the cell resistance with a final *R*_cl_ ~1.5-fold higher than the starting resistance before the wound. This can be attributed to the formation of a densely packed healing front (less permeable to ions/higher resistance) due to the incorporation of debris from the dead cells lying on top the transistor channel. This is clearly visible in the brightfield images collected 1.5 and 3 h after starting of the healing process as indicated by the red arrows (see Supplementary Video 3 ESI).

**Figure 5:**
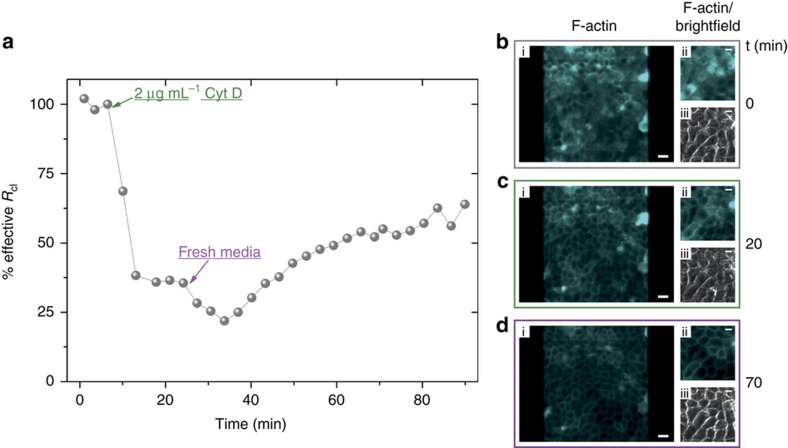
(**a**) Variation of the cell layer resistance of MDCK II-pLifeAct when exposed to cell culture media containing 2 μg mL^−1^ of cytochalasin D (Cyt D). A large drop in the cell-barrier resistance is visible within the first 10 min upon exposure of the cells to Cyt D. When fresh media (Cyt D free) was re-perfused inside the microchannel, a recovery of cell layer resistance was observed. On the right, the fluorescence images of the cells (**b**) before (gray frame), (**c**) during (green frame), and (**d**) after (purple frame) exposure to Cyt D are shown (scale bar, 20 μm). On the far right of the three frames zoom-in fluorescence and brightfield images of the central area of the transistor channel covered with the confluent cell layer (scale bar, 10 μm) are shown.

As a final demonstration of the platform for drug discovery/toxicology monitoring, cells were treated with Cyt D. The latter is part of a class of fungal metabolites that inhibit actin polymerization and also induce F-actin depolymerization in the cell cytoskeleton^59^. As Cyt D affects the actin filaments of the cells, we monitored its effect on MDCK II-pLifeAct cells using the combined optical and electronic monitoring system. Similar to the previous section, cells were grown for 3 days at 1.67 μL min^−1^ to reach confluency and expression of appropriate cell-barrier properties. A DMEM media solution containing 2 μg mL^−1^ of Cyt D was perfused in the microchannel while both brightfield and fluorescence images were collected and *R*_cl_ monitored. Cyt D was incubated with the cells under static conditions. The fluorescence image at time zero of Figure 5bi (gray frame) shows a confluent layer of cells uniformly covering the transistor channel area (200×200 μm^2^). Figure 5bii and iii shows a zoom of fluorescence and brightfield images of the cell layer covering the central area of the transistor channel. Under these conditions, the cells show a diffuse staining of actin. As the Cyt D starts to disrupt the actin cytoskeleton (see Supplementary ESI), we observed an abrupt drop of the cell resistance, that is, 60% decrease in less than 5 min, as shown in Figure 5a. The fluorescence image of Figure 5ci captured at *t*=20 min shows some changes in the distribution of the actin in the cells, with the fluorescent actin now predominantly on the borders of the cells, more clearly visible in Figure 5cii (green frame). Optical observations are relatively subjective, and the extent of the barrier damage caused by the presence of Cyt D is difficult to quantify, especially when comparing the two brightfield images at *t*=0 of Figure 5bii and *t*=20 min of Figure 5cii. In contrast, impedance monitoring using the OECTs offers a highly sensitive label-free tool to study cell/drug interaction dynamics in a more exhaustive, direct, and quantifiable manner. In addition, we were also interested to ascertain the dynamics on the recovery of the barrier properties when the Cyt D is removed using the microfluidics, as shown in Figure 5a. As fresh cell culture media was perfused inside the microchannel, we first monitored a further 20% decrease of the cell resistance. The drop in *R*_cl_ can be attributed to the mechanical movements of the cells caused by the initial perfusion of new fresh media in the microchannel. In fact, as Cyt D acts on the polymerization of the actin filaments, we believe that cells lose their plasticity and ability to support any external mechanical stress, that is, liquid shear. However, following this initial drop in the cell resistance, a gradual increase in the *R*_cl_ is observed as actin polymerization in the cell cytoskeleton can resume and cells can recover their healthy barrier properties. Figure 5d shows images captured at *t*=70 min, in which the fluorescent actin is predominantly at the periphery of the cells.

Finally, we also wanted to test the effect that a short exposure (20 min) to Cyt D can have on the healing of cells. As Cyt D inhibits the actin polymerization, its presence should adversely influence the healing process as cells should not be able to move due to the depolymerization of the actin filament in the cell cytoskeleton. As expected, electrically wounded cells exposed to Cyt D were not able to form a healing front, and thus heal, when cells were monitored optically and electronically for over 10 h (see Supplementary ESI). Nevertheless, a completely healed wound was only observed 24 h after the beginning of the healing process.

## Conclusions

In conclusion, we have developed a simple strategy to integrate a microfluidic platform with a highly sensitive label-free sensor based on the OECT technology. With the expansion of the organ-on-a-chip field, there is an urgent need for the coupling of these model organs with more reliable in-line sensors capable of providing real-time, multi-parametric information on cell integrity. Since *in vitro* models are increasingly being designed for operation in the range of days to weeks, this poses a significant challenge for adapted monitoring systems. Currently, many organ-on-chip models are only assessed at the end of the experiment, usually by disassembly of the device, in a so-called end-point assay^10^. In the literature, few examples of impedance electrical monitoring integrated with organ-on-chip devices have been described^19,20^, and the electrode materials employed, for example, silver chloride (AgCl), are known to be a significant source of cytotoxicity if used for anything other than chronic measurements^60^. In a recent study of an *in vitro* model of the gastrointestinal–microbe interface, commercially available chopstick electrodes were employed for the measurement of the cell TER^16^. However, the insertion of these electrodes lead to the contamination of the microfluidic platform, thus limiting its employment as a true in-line sensing system, to say nothing of the issues related to irreproducibility of chopstick electrode readings^32^. In literature, other kidney-on-a-chip type of device have been already reported^10,61,62^, but except from the work of Ferrell *et al.*^62^ their integration with in-line label-free sensors was not explored yet. For the latter, however, manual positioning of Ag/AgCl and silver wires in the microfluidics was needed to locate the recording electrode inside the bioreactor. In comparison here, we have made use of planar microfabricated OECTs and an easy strategy for their integration inside the proposed microfluidic platform.

Dual advantages of the use of the OECT used as a multi-parametric in-line sensor are the biocompatibility of the PEDOT:PSS active layer (related to hydrogel-like properties)^27^ as well as a flexibility to customize the transistor configuration, for example, planar or vertical geometry with regard to the requirements of the *in vitro* model under investigation. Integration of the devices on flexible substrates has already been demonstrated^29^ and 3D electrode formats are in progress^63,64^. Here we demonstrated the use of the OECT impedance sensor for the extraction of the cell layer resistance and capacitance in real time while the epithelium was stimulated by the biomechanical action of the fluid flow. As we have demonstrated previously, electronic monitoring provides more sensitive and quantitative information on conformational and structural changes occurring to the cells compared to standard end-point assays, such as immunofluorescence. An important feature of our platform is the ability to carry out simultaneous optical monitoring, which validates our data while providing a benchmark for scientists used to microscopy-based techniques. The ability to monitor key metabolites using the OECT is an added benefit, and direct, *in situ* monitoring of molecules such as glucose and lactate will be featured in future devices. A final addition to the multi-parametric repertoire is the development of a fully automated electrical wound-healing assay, taking advantage of the transparent nature of the transistor active layer allowing us to perform live electrical and optical monitoring of the healing process as it occurred.

In this study, we clearly demonstrate the ability to readily embed in-line sensors in microfluidics, representing the first step toward a truly integrated platform for the realization of high-content, high-throughput *in vitro* testing. Future projects will concentrate on harnessing the literal flexibility of the conducting polymer devices shown here for the development of other platforms, such as multilayer microfluidics, to mimic basal and apical compartments of the cells as well as integration of 3D electroactive polymer scaffolds.

## References

[bib1] Hartung T. Food for thought look back in anger–What clinical studies tell us about preclinical work. Altex 2013; 30: 275–291. 23861075 10.14573/altex.2013.3.275 PMC3790571

[bib2] Avior Y, Sagi I, Benvenisty N. Pluripotent stem cells in disease modelling and drug discovery. Nature Review Molecular Cell Biology 2016; 17: 170–182. 26818440 10.1038/nrm.2015.27

[bib3] McKim JM. Building a tiered approach to *in vitro* predictive toxicity screening: A focus on assays with *in vivo* relevance. Combinatorial Chemistry & High Throughput Screening 2010; 13: 188–206. 20053163 10.2174/138620710790596736 PMC2908937

[bib4] Breslin S, O’Driscoll L. Three-dimensional cell culture: The missing link in drug discovery. Drug Discovery Today 2013; 18: 240–249. 23073387 10.1016/j.drudis.2012.10.003

[bib5] Elliott NT, Yuan F. A review of three-dimensional *in vitro* tissue models for drug discovery and transport studies. Journal of Pharmaceutical Sciences 2011; 100: 59–74. 20533556 10.1002/jps.22257

[bib6] Pampaloni F, Reynaud EG, Stelzer EHK. The third dimension bridges the gap between cell culture and live tissue. Nature Review Molecular Cell Biology 2007; 8: 839–845. 17684528 10.1038/nrm2236

[bib7] Bhatia SN, Ingber DE. Microfluidic organs-on-chips. Nature Biotechnology 2014; 32: 760–772. 10.1038/nbt.2989 25093883

[bib8] Esch EW, Bahinski A, Huh D. Organs-on-chips at the frontiers of drug discovery. Nature Reviews Drug Discovery 2015; 14: 248–260. 25792263 10.1038/nrd4539 PMC4826389

[bib9] Stucki AO, Stucki JD, Hall SRR et al. A lung-on-a-chip array with an integrated bio-inspired respiration mechanism. Lab on a Chip 2015; 15: 1302–1310. 25521475 10.1039/c4lc01252f

[bib10] Jang K-J, Mehr AP, Hamilton GA et al. Human kidney proximal tubule-on-a-chip for drug transport and nephrotoxicity assessment. Integrative Biology 2013; 5: 1119–1129. 23644926 10.1039/c3ib40049b

[bib11] Maschmeyer I, Lorenz AK, Schimek K et al. A four-organ-chip for interconnected long-term co-culture of human intestine, liver, skin and kidney equivalents. Lab on a Chip 2015; 15: 2688–2699. 25996126 10.1039/c5lc00392j

[bib12] Abaci HE, Gledhill K, Guo Z et al. Pumpless microfluidic platform for drug testing on human skin equivalents. Lab on a Chip 2015; 15: 882–888. 25490891 10.1039/c4lc00999a PMC4305008

[bib13] Kolesky DB, Homan KA, Skylar-Scott MA et al. Three-dimensional bioprinting of thick vascularized tissues. Proceedings of the National Academy of Sciences of the United States of America 2016; 113: 3179–3184. 26951646 10.1073/pnas.1521342113 PMC4812707

[bib14] Zhang W, Zhang YS, Bakht SM et al. Elastomeric free-form blood vessels for interconnecting organs on chip systems. Lab on a Chip 2016; 16: 1579–1586. 26999423 10.1039/c6lc00001k PMC4846563

[bib15] Huval RM, Miller OH, Curley JL et al. Microengineered peripheral nerve-on-a-chip for preclinical physiological testing. Lab on a Chip 2015; 15: 2221–2232. 25850799 10.1039/c4lc01513d

[bib16] Shah P, Fritz JV, Glaab E et al. A microfluidics-based *in vitro* model of the gastrointestinal human-microbe interface. Nature Communications 2016; 7: 11535. 10.1038/ncomms11535 PMC4865890 27168102

[bib17] Miura S, Sato K, Kato-Negishi M et al. Fluid shear triggers microvilli formation via mechanosensitive activation of TRPV6. Nature Communications 2015; 6: 8871. 10.1038/ncomms9871 PMC4660203 26563429

[bib18] Zheng Y, Chen J, López JA. Flow-driven assembly of VWF fibres and webs in *in vitro* microvessels. Nature Communications 2015; 6: 7858. 10.1038/ncomms8858 PMC4522708 26223854

[bib19] Esch MB, Ueno H, Applegate DR et al. Modular, pumpless body-on-a-chip platform for the co-culture of GI tract epithelium and 3D primary liver tissue. Lab on a Chip 2016; 16: 2719–2729. 27332143 10.1039/c6lc00461j

[bib20] Booth R, Kim H. Characterization of a microfluidic *in vitro* model of the blood-brain barrier (μBBB). Lab on a Chip 2012; 12: 1784–1792. 22422217 10.1039/c2lc40094d

[bib21] Perestrelo AR, Águas ACP, Rainer A et al. Microfluidic organ/body-on-a-chip devices at the convergence of biology and microengineering. Sensors 2015; 15: 31142–31170. 26690442 10.3390/s151229848 PMC4721768

[bib22] Huh D, Hamilton GA, Ingber DE. From three-dimensional cell culture to organs-on-chips. Trends in Cell Biology 2011; 21: 745–754. 22033488 10.1016/j.tcb.2011.09.005 PMC4386065

[bib23] Rivnay J, Owens RM, Malliaras GG. The rise of organic bioelectronics. Chemistry of Materials 2014; 26: 679–685.

[bib24] Simon DT, Gabrielsson EO, Tybrandt K et al. Organic bioelectronics: Bridging the signaling gap between biology and technology. Chemical Reviews 2016; 116: 13009–13041. 27367172 10.1021/acs.chemrev.6b00146

[bib25] Khodagholy D, Rivnay J, Sessolo M et al. High transconductance organic electrochemical transistors. Nature Communications 2013; 4: 2133. 10.1038/ncomms3133 PMC3717497 23851620

[bib26] Strakosas X, Sessolo M, Hama A et al. A facile biofunctionalisation route for solution processable conducting polymer devices. Journal of Material Chemistry B 2014; 2: 2537–2545. 10.1039/c3tb21491e 32261421

[bib27] Strakosas X, Wei B, Martin DC et al. Biofunctionalization of polydioxythiophene derivatives for biomedical applications. Journal of Material Chemistry B 2016; 4: 4952–4968. 10.1039/c6tb00852f 32264022

[bib28] Khodagholy D, Curto VF, Fraser KJ et al. Organic electrochemical transistor incorporating an ionogel as a solid state electrolyte for lactate sensing. Journal of Material Chemistry 2012; 22: 4440–4443.

[bib29] Khodagholy D, Doublet T, Quilichini P et al. *In vivo* recordings of brain activity using organic transistors. Nature Communications 2013; 4: 1575. 10.1038/ncomms2573 PMC3615373 23481383

[bib30] Pappa A-M, Curto VF, Braendlein M et al. Organic transistor arrays integrated with finger-powered microfluidics for multianalyte saliva testing. Advanced Healthcare Materials 2016; 17: 2295–2302. 10.1002/adhm.201600494 27385673

[bib31] Sessolo M, Rivnay J, Bandiello E et al. Ion-selective organic electrochemical transistors. Advanced Materials 2014; 26: 4803–4807. 24862110 10.1002/adma.201400731

[bib32] Jimison LH, Tria SA, Khodagholy D et al. Measurement of barrier tissue integrity with an organic electrochemical transistor. Advanced Materials 2012; 24: 5919–5923. 22949380 10.1002/adma.201202612

[bib33] Ramuz M, Hama A, Rivnay J et al. Monitoring of cell layer coverage and differentiation with the organic electrochemical transistor. Journal of Material Chemistry B 2015; 3: 5971–5977. 10.1039/c5tb00922g 32262653

[bib34] Yao C, Li Q, Guo J et al. Flexible organic electrochemical transistor arrays for monitoring action potentials from electrogenic cells. Advanced Healthcare Materials 2015; 4: 528–533. 25358525 10.1002/adhm.201400406

[bib35] Ramuz M, Hama A, Huerta M et al. Combined optical and electronic sensing of epithelial cells using planar organic transistors. Advanced Materials 2014; 26: 7083–7090. 25179835 10.1002/adma.201401706 PMC4489338

[bib36] Shim NY, Bernards DA, Macaya DJ et al. All-plastic electrochemical transistor for glucose sensing using a ferrocene mediator. Sensors 2009; 9: 9896–9902. 22303153 10.3390/s91209896 PMC3267201

[bib37] Strakosas X, Huerta M, Donahue MJ et al. Catalytically enhanced organic transistors for *in vitro* toxicology monitoring through hydrogel entrapment of enzymes. Journal of Applied Polymer Science 2016; 134, 44483; doi: 10.1002/app.44483.

[bib38] Braendlein M, Pappa A-M, Ferro M et al. Lactate detection in tumor cell cultures using organic transistor circuits. Advanced Materials 2017; 29: 1605744. 10.1002/adma.201605744 28134450

[bib39] DeFranco JA, Schmidt BS, Lipson M et al. Photolithographic patterning of organic electronic materials. Organic Electronics 2006; 7: 22–28.

[bib40] Rivnay J, Ramuz M, Leleux P et al. Organic electrochemical transistors for cell-based impedance sensing. Applied Physics Letters 2015; 106: 043301.

[bib41] Tan CP, Craighead HG. Surface engineering and patterning using parylene for biological applications. Materials 2010; 3: 1803–1832.

[bib42] Tang L, Lee NY. A facile route for irreversible bonding of plastic-PDMS hybrid microdevices at room temperature. Lab on a Chip 2010; 10: 1274–1280. 20445880 10.1039/b924753j

[bib43] Wu W, Wu J, Kim J-H et al. Instantaneous room temperature bonding of a wide range of non-silicon substrates with poly(dimethylsiloxane) (PDMS) elastomer mediated by a mercaptosilane. Lab on a Chip 2015; 15: 2819–2825. 26014886 10.1039/c5lc00285k

[bib44] Curto VF, Fay C, Coyle S et al. Real-time sweat pH monitoring based on a wearable chemical barcode micro-fluidic platform incorporating ionic liquids. Sensors and Actuators B: Chemical 2012; 171–172: 1327–1334.

[bib45] Jang K-J, Suh K-Y. A multi-layer microfluidic device for efficient culture and analysis of renal tubular cells. Lab on a Chip 2010; 10: 36–42. 20024048 10.1039/b907515a

[bib46] Young EWK, Beebe DJ. Fundamentals of microfluidic cell culture in controlled microenvironments. Chemical Society Reviews 2010; 39: 1036. 20179823 10.1039/b909900j PMC2967183

[bib47] Duan Y, Gotoh N, Yan Q et al. Shear-induced reorganization of renal proximal tubule cell actin cytoskeleton and apical junctional complexes. Proceedings of the National Academy of Sciences of the United States of America 2008; 105: 11418–11423. 18685100 10.1073/pnas.0804954105 PMC2516248

[bib48] Brown JW, McKnight CJ. Molecular model of the microvillar cytoskeleton and organization of the brush border. PLoS ONE 2010; 5: 9406. 10.1371/journal.pone.0009406 PMC2827561 20195380

[bib49] DePaola N, Phelps JE, Florez LK et al. Electrical impedance of cultured endothelium under fluid flow. Annals of Biomedical Engineering 2001; 29: 648–656. 11556721 10.1114/1.1385811

[bib50] Benson K, Cramer S, Galla H-J. Impedance-based cell monitoring: barrier properties and beyond. Fluids Barriers CNS 2013; 10: 5. 23305242 10.1186/2045-8118-10-5 PMC3560213

[bib51] Srinivasan B, Kolli AR, Esch MB et al. TEER measurement techniques for *in vitro* barrier model systems. Journal of Laboratory Automation 2015; 20: 107–126. 25586998 10.1177/2211068214561025 PMC4652793

[bib52] Pappa A-M, Curto VF, Braendlein M et al. Organic transistor arrays integrated with finger-powered microfluidics for multianalyte saliva testing. Advanced Healthcare Materials 2016; 5: 2295–2302. 27385673 10.1002/adhm.201600494

[bib53] Lange K. Role of microvillar cell surfaces in the regulation of glucose uptake and organization of energy metabolism. American Journal of Physiology – Cell Physiology 2002; 282: 21–26. 10.1152/ajpcell.2002.282.1.C1 11742794

[bib54] Keese CR, Wegener J, Walker SR et al. Electrical wound-healing assay for cells *in vitro*. Proceedings of the National Academy of Sciences of the United States of America 2004; 101: 1554–1559. 14747654 10.1073/pnas.0307588100 PMC341773

[bib55] Wegener J, Keese CR, Giaever I. Recovery of adherent cells after *in situ* electroporation monitored electrically. BioTechniques 2002; 33: 348. 12188187 10.2144/02332rr01

[bib56] Kiehart DP. Wound healing: The power of the purse string. Current Biology 1999; 9: 602–605. 10.1016/s0960-9822(99)80384-4 10469588

[bib57] Teissie J, Golzio M, Rols MP. Mechanisms of cell membrane electropermeabilization: A minireview of our present (lack of ?) knowledge. Biochimica Et Biophysica Acta (BBA)-General Subjects 2005; 1724: 270–280. 15951114 10.1016/j.bbagen.2005.05.006

[bib58] Tehrani P, Kanciurzewska A, Crispin X et al. The effect of pH on the electrochemical over-oxidation in PEDOT:PSS films. Solid State Ionics 2007; 177: 3521–3527.

[bib59] Casella JF, Flanagan MD, Lin S. Cytochalasin D inhibits actin polymerization and induces depolymerization of actin filaments formed during platelet shape change. Nature 1981; 293: 302–305. 7196996 10.1038/293302a0

[bib60] Loza K, Sengstock C, Chernousova S et al. The predominant species of ionic silver in biological media is colloidally dispersed nanoparticulate silver chloride. RSC Advances 2014; 4: 35290.

[bib61] Ferrell N, Desai RR, Fleischman AJ et al. A microfluidic bioreactor with integrated transepithelial electrical resistance (TEER) measurement electrodes for evaluation of renal epithelial cells. Biotechnology and Bioengineering 2010; 107: 707–716. 20552673 10.1002/bit.22835 PMC3903011

[bib62] Sakolish CM, Mahler GJ. A novel microfluidic device to model the human proximal tubule and glomerulus. RSC Advances 2017; 7: 4216–4225.

[bib63] Wan AM-D, Inal S, Williams T et al. 3D conducting polymer platforms for electrical control of protein conformation and cellular functions. Journal of Materilas Chemistry B 2015; 3: 5040–5048. 10.1039/C5TB00390C PMC4582673 26413300

[bib64] Inal S, Hama A, Ferro M et al. Conducting Polymer Scaffolds for Monitoring 3D Cell Culture. Advanced Biosystems 2017; 1: 1700052; http://dx.doi.org/10.1002/adbi.201700052.

