# Supplementary material for: Organic transistor platform with integrated microfluidics for in-line multi-parametric in vitro cell monitoring
Source: Microsyst Nanoeng. 2017 Aug 14;3:17028. doi: 10.1038/micronano.2017.28 (PMC6445009; doi:10.1038/micronano.2017.28)
Supplement: Supplementary Information [file micronano201728-s1.pdf]

Supporting file

Organic transistor platform with integrated microfluidics for in-line multi-parametric *in vitro* cell monitoring

Vincenzo F. Curto[1], *Bastien Marchiori*[1,2], *Adel Hama*[1], *Magali P. Ferro*[1],*Anna-Maria Pappa*[1], *Marcel Braendlein*[1], *Jonathan Rivnay*[1,*], *Michel Fiocchi* [1], *George G. Malliaras*[1], *Marc Ramuz*[2], *Róisín M. Owens*[1]

[1]Department of Bioelectronics, Ecole Nationale Superieure des Mines, CMP-EMSE, MOC, 880 avenue de Mimet, Gardanne 13541, France
[2]Flexible Electronics Department, Ecole Nationale Supérieure des Mines CMP-EMSE, MOC, 880 avenue de Mimet 13541, Gardanne, France
[*]present address: Dept. of Biomedical Engineering, Northwestern University, Evanston, IL 60208, USA

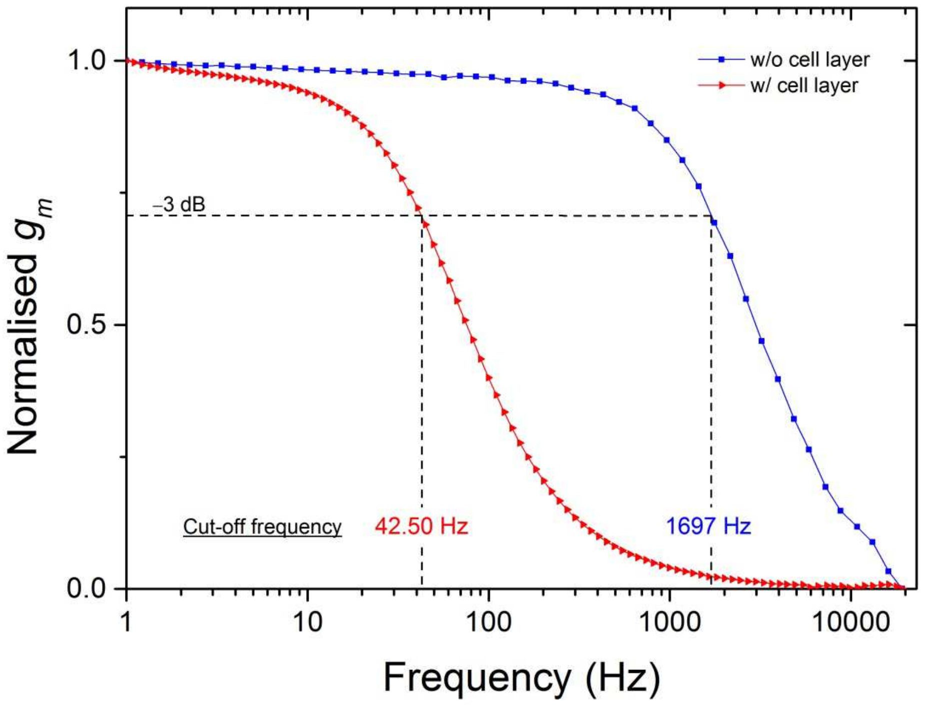

Figure S1. Frequency-dependent response of an organic electrochemical transistor in the absence and presence of a confluent cell layer. Typical frequency-dependent OECT response in the absence (blue squares) and presence of a confluent layer of MDCK II cells (red triangles) covering the transistor channel area (50 x 50 µm²). In the absence of cells, the transconductance (g_m) presents a plateau until approximately 1000 Hz and then it drops abruptly with a cut-off value of 1697 Hz. When a fully confluent cell layer of MDCK II is grown on top of the transistor channel, a decrease in the cut-off frequency is observed (42.50 Hz) due to the presence of cells.[1]

An OECT was used as an electrochemical biosensor for the enzymatic determination of glucose in MDCKII cell culture media. Specifically, the planar gate electrode of the transistor was functionalized based on a previously reported method.[2] Briefly, the enzyme glucose oxidase (GO_x) was immobilized on the PEDOT:PSS gate together with an electrochemical mediator (Chitosan-Ferrocene, CS-Fc) complex. A more detailed explanation of the gate biofunctionalization is provided in the Experimental Section. The use of the mediator enabled us to lower the operation potential, thereby minimizing any electrochemical interference due to oxidation of the inherently electroactive species present in the cell culture media. The sensing mechanism of our platform is based on the electron shuttling from the reduced enzyme (upon contact with the analyte) to the PEDOT:PSS electrode by the ferrocene/ferricenium (Fc/Fc^+) ion couple. This results in a change in the gating of the channel proportional to the analyte concentration thereby allowing its quantification. The transistor's channel geometry (Width/ Length, W/L =10/100 μm and thickness, d= 90 nm) was customized to exhibit highest transconductance value at the gate potential of 0.2 V, to coincide with the working potential of our enzymatic sensors. Prior to the measurements, a calibration curve of glucose in the MDCK II cell media was performed (**Figure S2**) to determine the appropriate media dilution (maximum value in the linear range) for maximum sensitivity and accuracy. All characterizations were performed using 1X PBS. The chronoamperometric measurements of the OECTs were recorded using a Keithley 2612A dual SourceMeter with customized LabVIEW software. For the measurements, the gate potential V_G was kept constant at 0.2 V while the source-drain potential V_{SD} at – 0.6 V.

The device response for each dilution was obtained from each steady current level and normalized according to the following equation:

$$NR_x = |(I_C - I_{C0})/ I_{C0}| \quad (1)$$

where NR_x is the normalise response at a specific analyte concentration, I_{C0}, I_C are the current outputs in the absence and in the presence of the target analyte, respectively.

For the glucose uptake measurements, the cell culture media samples were collected from the microfluidic device over time (before and after 20 μL min^{-1}flow rate as well as in the control condition of 1.6 μL min^{-1} flow rate) and stored at – 4°C after collection. All the samples were diluted in 1X PBS (1:5) and glucose content determination was then performed. Prior and post measurements, the bionsensor was calibrated in order to verify possible degradation in the enzymatic response. Percentage of the glucose uptake was determined from the following equation:

$$\text{Glucose uptake \% } NR_x = |1 - NR_x| * 100 \quad (2)$$

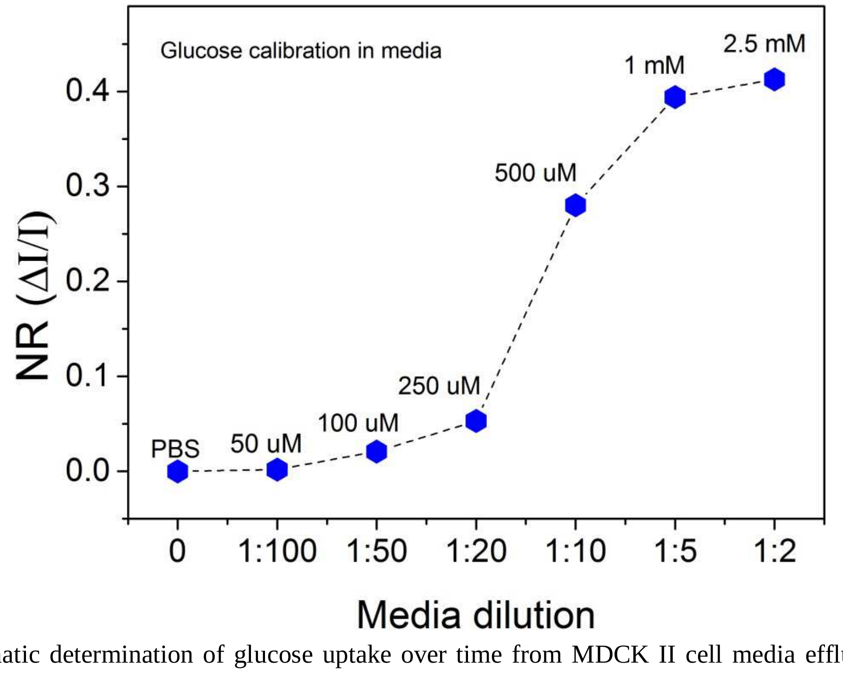

Figure S2. Enzymatic determination of glucose uptake over time from MDCK II cell media effluent. Normalized calibration curve obtained from the chronoamperometric response of the GOx/CSFc functionalized OECTs (channel dimension *W*=100 μm and *L*=10 μm) using different dilution ratio of PBS (pH 7.4, 1X) and fresh DMEM media. The reported concentration values at each data point are the final concentration of glucose resulting from the dilution ratio, as initial glucose concentration in DMEM cell culture media (Gibco) is reported to be 5 mM. 1:5 dilution was chosen as the optimum operation condition for the assay of the microfluidics effluent specimens in order to operate the sensor at its highest sensitivity.

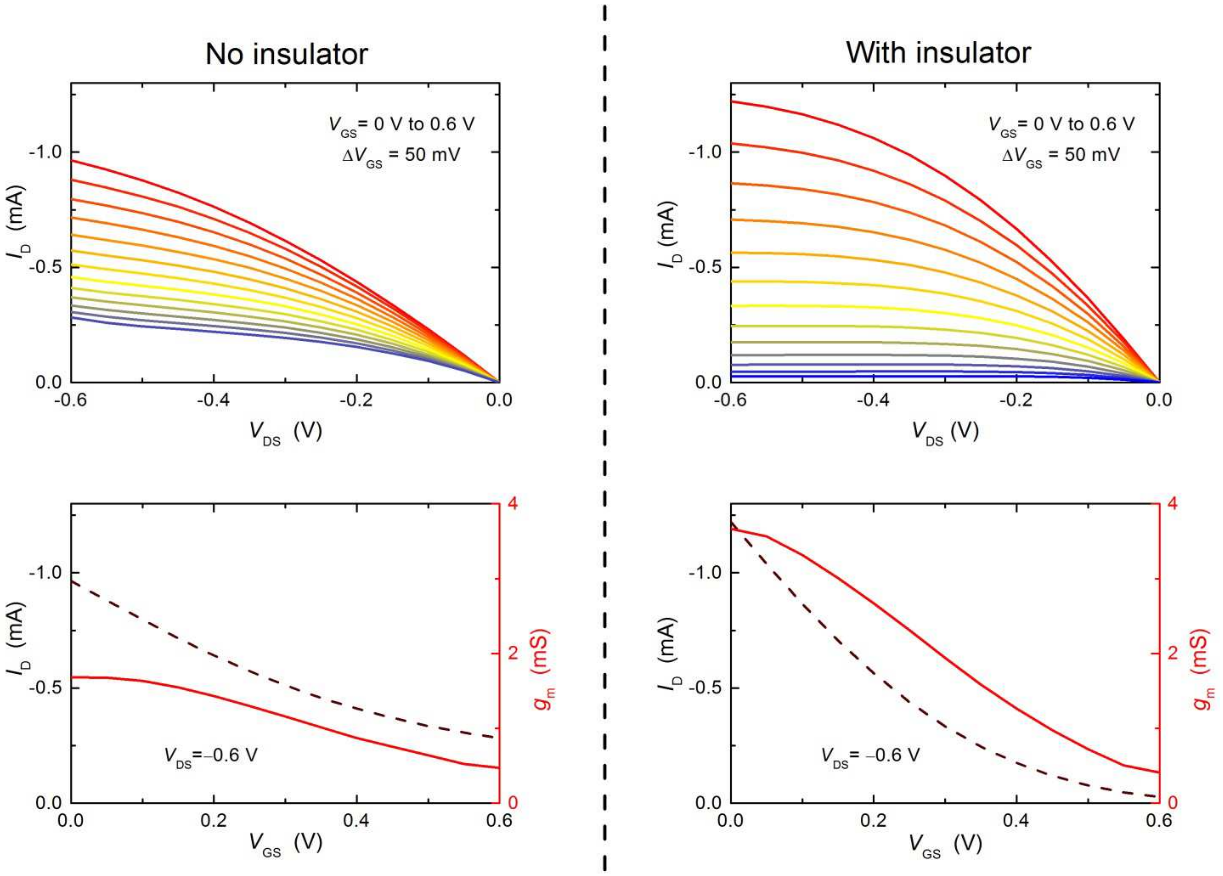

Figure S3. Influence of the presence of the parylene C (PaC) insulating layer on the organic electrochemical transistor (OECT) performance. Typical output- and transfer-curves for an OECT device without (left) and with (right) insulating PaC layer covering the gold leads. The dimensions of the active area are W = 50 µm, L = 50 µm, and d = 70 nm. It can be seen that the device without insulation cannot be fully de-doped even at high positive gate voltages. Furthermore, the transconductance and hence the sensitivity of the sensor is considerably lower. The peak transconductance drops by a factor of 2.3 as compared to a device with insulation. We believe this to be attributed to an additional capacitive layer forming at the electrolyte-gold interface which alters the electrostatic properties of the OECT. The cations accumulating at the negatively biased drain contact might locally hinder cations from being pushed into the conducting polymer by to the positive gate bias. With the insulation, this effect is minimized and optimal behaviour of the OECT can be achieved.

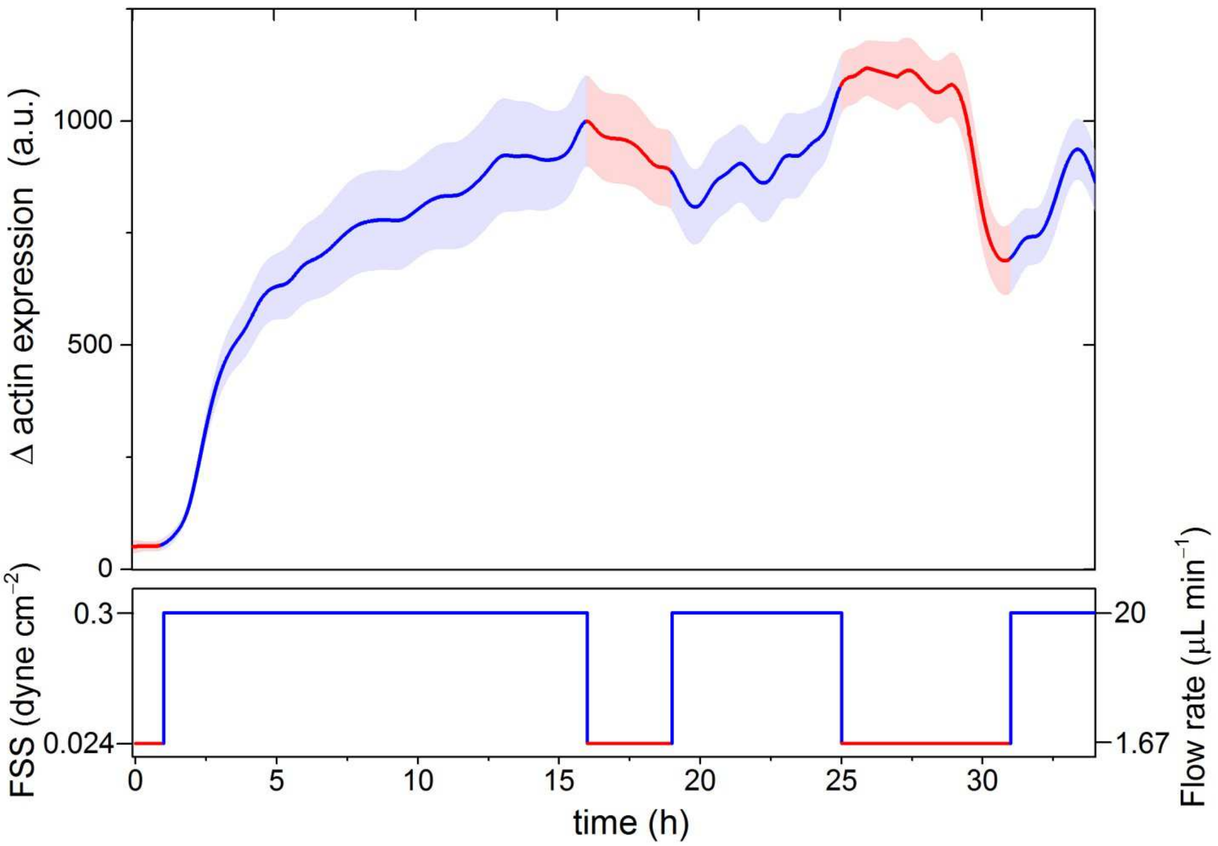

Figure S4. Cyclical variation of the F-actin expression induced by the increase and decrease of the flow rate. Cyclical variations in the cell F-actin expression is induced by varying the fluid shear stress (FSS) in the microfluidic channel, shown here as the relative increase in the fluorescence intensity (λ=584 nm). Cells are grown to confluency under dynamic conditions with a flow rate equal to 1.67 μL min^{-1}, until cells show a typical cobblestone-like morphology. The epithelial cells (MDCK II pLifeAct) are then mechanically stimulated with a physiologically relevant FSS equal to 0.3 dyne cm^{-2} for 15 hours. Once the physiologically relevant FSS was stopped for 3 hours a decrease in the F-actin fluorescence was observed. A subsequent increase in the F-actin was measured following 6 more hours at 0.3 dyne cm^{-2} of FSS stimulation. A similar trend in F-Actin fluorescence variation was observed when a second flow rate/FSS cycle was repeated this time for 6 hours, also illustrating the time dependence of the flow cycles. The FSS/flow rate profile used for the experiment is also represented.

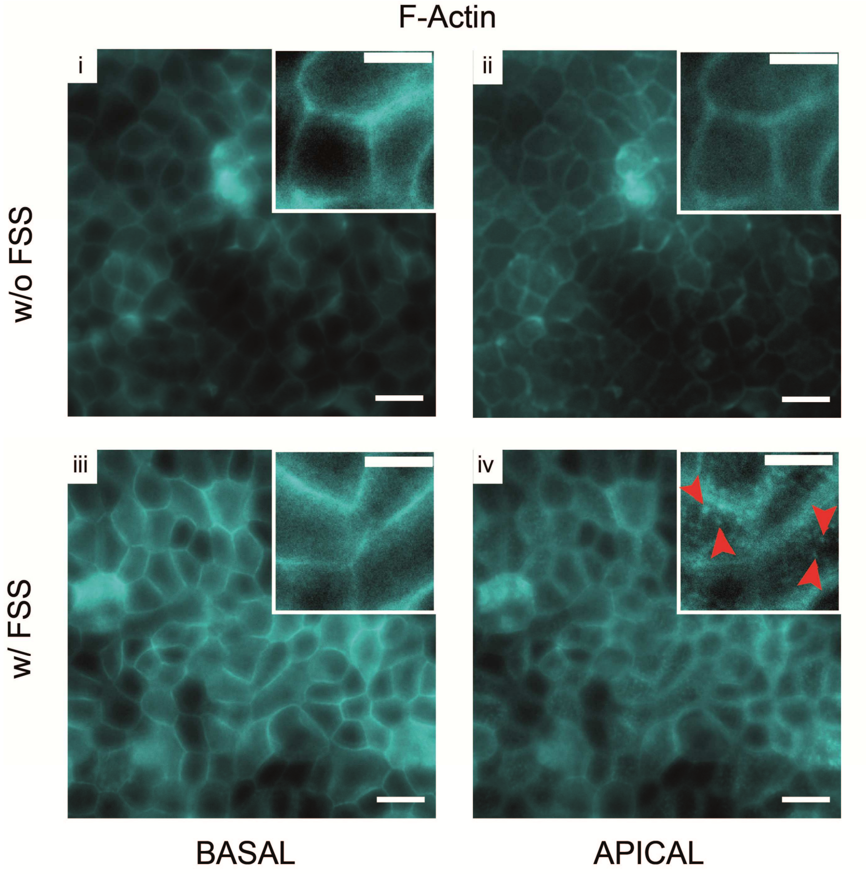

Figure S5. F-actin fluorescence images from the apical and basal side of a confluent cell layer of MDCKII-pLifeAct with and without FSS stimulation. FSS stimulated cells show the presence of highly fluorescent dots (red arrow from the inset iv) posited to be due to the formation of microvilli structures on the apical side of the cell layer. No such structures were visible on the apical side of the cells that were grown in the microfluidic device without stimulation by a physiologically relevant FSS, Figure S3-i and –ii. (scale bar 20 µm (10 µm for inset images)).

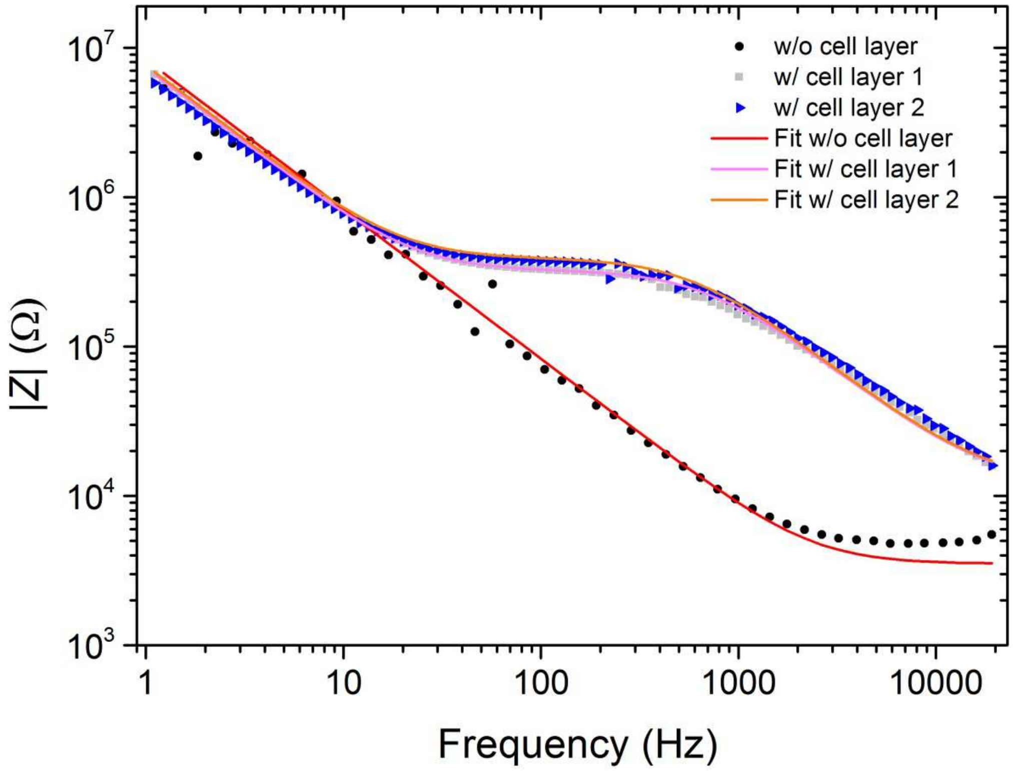

Figure S6. Measured and fitted impedance spectra in the presence and absence of a confluent cell layer covering the OECT channel. Representative impedance spectra recorded with and without a confluent layer lining the bottom substrate of the microfluidics/OECT platform. Black dots and red line show the measured data points and the fitting model [$R_s(R_{OECT}C_{OECT})$], respectively, when no cells are covering the transistor. In the presence of a confluent layer of cells, the impedance spectrum shows a plateau region (f(Hz) ≤ 400 Hz) attributed to the paracellular resistance of the cell covering the active area of the device. In the figure two separate set of data are reported (grey square and blue triangle) recorded during and after FSS stimulation in microfluidics, respectively. The equivalent fitting curves calculated using an equivalent circuit model, previously reported by us,[1] show a discrete difference in the plateau region below 400 Hz attributed to changes in the cell layer resistance R_{cl}.

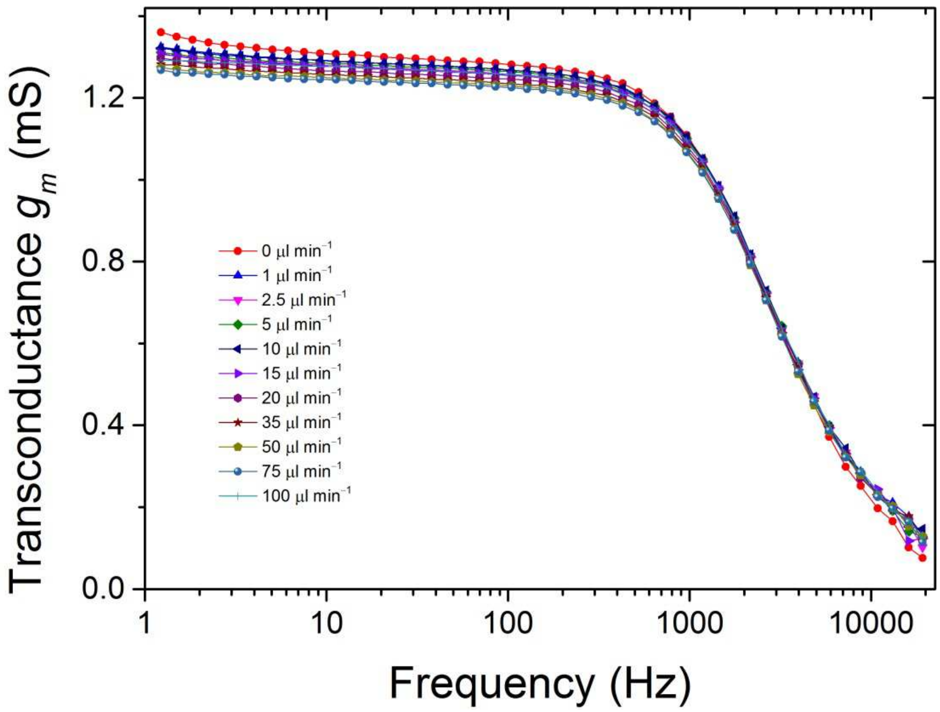

Figure S7. Influence of different flow rates in microfluidics on the frequency dependent OECT response. Series of bandwidth spectra measured in the absence of a cell layer lining the microfluidic channel in order to ascertain any possible influence on the liquid motion on the OECT response. The measurements were performed using as the electrolyte cell culture media (Advanced DMEM Reduced Serum Medium 1, Invitrogen) perfused inside the microchannel at different flow rates, ranging from 0 to 100 µL min^{-1}. A slight decrease in the maximum transconductance was observed (plateau region), however no significant changes in the OECT response could be detected. The OECT geometry is 50 x 50 µm^2.

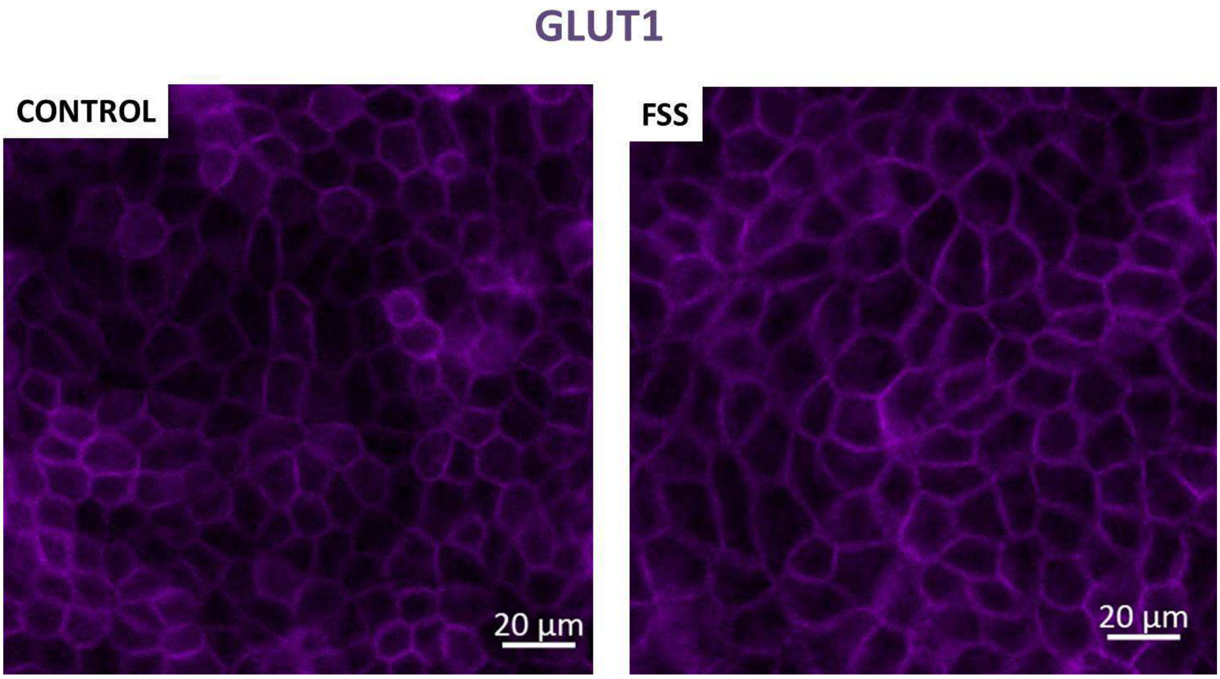

Figure S8. GLUT1 cell membrane glucose transporter immunofluorescence images. Fluorescence images for immunofluorescence stained GLUT1 membrane glucose transporter. Cells are grown to confluency under dynamic conditions with a flow rate equal to 1.6 µL min^{-1}. In the control condition, cells were kept in culture with a constant flow rate of 1.6 µL min^{-1} before being fixed and labelled. For the FSS (fluid shear stress) conditions, once the epithelium is fully confluent, a greater flow rate of 20 µL min^{-1} is applied for 15 hours. Cells were fixed and labelled 2 hours after the FSS was stopped. Control and FSS cells present similar distribution of GLUT1 transporter in the cell membrane.

References

1. Rivnay, J. *et al.* Organic electrochemical transistors for cell-based impedance sensing. *Appl. Phys. Lett.* **106,** 043301 (2015).

2. Pappa, A.-M. *et al.* Organic transistor arrays integrated with finger-powered microfluidics for multianalyte saliva testing. *Adv. Healthc. Mater.* **5,** 2295–2302 (2016).
